# Reducing Efficiency of Fucoxanthin in Diatom Mediated Biofabrication of Gold Nanoparticles

**DOI:** 10.3390/ma14154094

**Published:** 2021-07-22

**Authors:** Piya Roychoudhury, Przemysław Dąbek, Michał Gloc, Aleksandra Golubeva, Renata Dobrucka, Krzysztof Kurzydłowski, Andrzej Witkowski

**Affiliations:** 1 Institute of Marine and Environmental Sciences, University of Szczecin, Mickiewicza 16a, 70-383 Szczecin, Poland; przemyslaw.dabek@usz.edu.pl (P.D.); alexandra.golubeva@phd.usz.edu.pl (A.G.); andrzej.witkowski@usz.edu.pl (A.W.); 2 Faculty of Materials Science and Engineering, Warsaw University of Technology, Wołoska 141, 02-507 Warsaw, Poland; michalgloc@wp.pl (M.G.); renata.dobrucka@ue.poznan.pl (R.D.); 3 Department of Non-Food Products Quality and Packaging Development, Institute of Quality Science, Poznań University of Economics and Business, Niepodległości 10, 61-875 Poznań, Poland; 4 Faculty of Mechanical Engineering, Białystok University of Technology, Wiejska 45c, 15-351 Białystok, Poland; k.kurzydlowski@pb.edu.pl

**Keywords:** diatom, fucoxanthin, gold nanoparticle, *Nanofrustulum shiloi*

## Abstract

In the present investigation, fucoxanthin—one of the major pigments in diatoms—has been extracted from *Nanofrustulum shiloi* SZCZM1342, and its reducing efficiency in the biogenesis of gold nanoparticles (GNPs) was checked. Fucoxanthin extracted from golden-brown cells of *N. shiloi* was compared to the healthy, growing biomass of *N. shiloi* and standard fucoxanthin after separate exposure to 25 mg L^−1^ aqueous hydrogen tetrachloroaurate solutions at room temperature. Isolated and standard fucoxanthin were found to be able to reduce gold ions within 12 h whereas, the whole biomass turned pink in color after 72 h of reaction. The synthesized particles were characterized by UV-vis spectroscopy, scanning electron microscopy (SEM) and transmission electron microscopy (TEM). UV–vis spectroscopy of purple-colored suspensions showed the absorption band at approximately 520–545 nm, indicating a strong positive signal for GNP synthesis. The SEM study revealed the deposition of GNPs on siliceous frustules of metal-treated diatom cells. The TEM analysis confirmed the GNPs synthesized by whole biomass are triangular, spherical and hexagonal in nature, whereas the particles produced by extracted and standard fucoxanthin are all spherical in nature. This study demonstrates the involvement of fucoxanthin in the reduction of gold ions and subsequent production of gold nanospheres.

## 1. Introduction

Biogenesis of biocompatible noble metal nanoparticles, such as gold [1,2,3,4,5], silver [6,7,8,9,10,11], platinum [12] and palladium [13], is a popular choice nowadays because it avoids the use of toxic reducing agents required in traditional chemical synthesis approaches. The main drawback in the green synthesis of nanoparticles is their resulting heterogeneity, i.e., the formation of differently shaped nanoparticles of variable size. The formation of varying shaped particles is very usual in green synthesis as different reducing agents, such as proteins, polysaccharides and pigments [14], work together. Particles with different shapes are not suitable in medical applications [15] because the heterogeneous products lead to unacceptable (variable, unreliable) results. Biosynthesis of nanoparticles with a definite shape is possible by using a single reducing agent [16]. Therefore, it is very important to identify the reducing agents within a cell and to check their efficacy in synthesizing nanoparticles in isolated conditions.

Microalgae have already been recognized as one of the best bioreagents for the biogenic production of GNP due to their high metal uptake capacity and fast growth rate [17,18,19]. Among the various microalgae, diatoms—the ‘Natural silica-nanofactories’—are an exceptional source of gold ions reduction since synthesized particles remain attached to the frustules. Frustules are the hard, outer covering of diatom cells with specific ornamentation and are made from nanosilica. Sometimes, the immobilization of metal nanoparticles on silica is required to maintain their catalytic activity [20,21,22,23]. In diatom-mediated nanoparticle production, nanosilica reduce particle aggregation, precipitation and can help to retain the catalytic activity of synthesized particles.

The synthesis of GNPs by *Nitzschia obtusa* and *Navicula minima* was first studied by Chakraborty et al. (2006) [17]. Composite nanoparticles, such as silica–gold and polysaccharide–gold, were synthesized by employing *Navicula atomus* and *Diadesmis gallica* [24]. The gold-silica [19] and silver-silica [25] nano-biocomposites produced by *Halamphora* sp. showed DNA binding affinity and fluorescent property, respectively. The marine diatom *Stephanopyxis turris* [26] and freshwater diatom *Eolimna* *minima* [27] were reported as potential strains to synthesize GNPs at the intracellular level. Biosilica obtained from diatoms have already been decorated by different nanoparticles containing silver [28], germanium [29], titanium dioxide [30] and iron oxide [31] to fabricate hybrid microparticles, which are considered more stable and are also very useful in photothermal therapy and multimodal imaging for cancer detection [24]. Therefore, diatoms are very attractive resources for the synthesis of metal-silica nanoconjugates, and these hybrid nanoparticles are very useful for applications ranging from bioelectronics to biomedicine.

The major light-harvesting pigments in diatoms are chlorophyll a, chlorophyll c and fucoxanthin [32]. Diatoms are golden brown in color as the relative amount of carotenoids is greater than the amount of chlorophyll [33]. Fucoxanthin is the main carotenoid that can transfer up to 60% of the energy to chlorophyll a in diatoms [34]. Fucoxanthin has a strong antioxidant property because of its unique structure [35,36]. The presence of epoxide, as well as allenic bonds and hydroxyl, carbonyl and carboxyl groups, in the polyene chain of fucoxanthin increases its electron-donating ability [37]. It is already reported by many authors that functional groups such as hydroxyl, carbonyl and carboxyl are involved in the reduction of metal ions [24]. Spherical-shaped silver nanoparticle production by extracted fucoxanthin from *Amphora* sp. was observed by Jena et al. (2014) [38]. This indicates that fucoxanthin is a very efficient reducing agent in synthesizing metal nanoparticles. No published report is available regarding the synthesis of GNP by extracted fucoxanthin.

Here, we report, for the first time, biocompatible spherical-shaped GNP production using extracted fucoxanthin from *N. shiloi*, one of the smallest diatom species known to date, which can occur off marine coasts in masses and form natural blooms [39]. An eco-friendly method has been described to synthesize triangle-shaped GNPs exploiting the whole biomass of *N. shiloi* with detailed characterization employing UV-vis spectroscopy, SEM, TEM and energy dispersive X-ray analysis (EDAX).

## 2. Materials and Methods

### 2.1. Extraction of Fucoxanthin

The chain diatom, *Nanofrustulum shiloi* (strain number SZCZM1342), was procured from the Diatom Culture Collection, University of Szczecin, Poland, and was grown at a salinity of 35‰ in the f/2 culture medium [40] at 18 °C under a 16:8 light: dark cycle, illuminated with 50 μmol photons m^−2^ s^−1^ of white light. The healthy cells of *N. shiloi* were centrifuged at 5000 rpm for 5 min, then rinsed with ddH_2_O and recollected by centrifugation.

Pellets (20 mg) were suspended in 1.5 mL of 70% ethanol for fucoxanthin extraction following the protocol of Wang et al. (2018) [41]. The mixture was incubated at 40 °C for 2 h in a thermo-mixer. Finally, the yellow-colored extract was separated by centrifugation at 8000 rpm for 10 min. The yellow extract was stored at −20 °C for further use. The whole procedure was conducted under dark conditions.

### 2.2. Biofabrication of GNP

In total, 1 mL ethanol extract (~12 µg of fucoxanthin) of *N. shiloi* was added to a 50 mL aqueous solution of 25 mg L^−1^ (pH 6) hydrogen tetrachloroaurate (HAuCl_4_.xH_2_O; SRL, MW 339.79). Then, 0.5 mg of standard fucoxanthin (Sigma-Aldrich) was dissolved in 5 mL of 70% ethanol. A total of 100 µL of a solution of standard fucoxanthin was also added to the 50 mL, 25 mg L^−1^ (pH 6) HAuCl_4_ solution separately to check the reducing efficacy of fucoxanthin. The experimental sets were kept under dark conditions as fucoxanthin is very light sensitive.

The selected strain, *N. shiloi* (100 mg fresh weight), was exposed to 100 mL of 25 mg L^−^^1^ Au^3+^ solution pH 4 for 72 h. A series of background experiments using different concentrations (5, 15, 25 and 35 mg L^−^^1^) and pH range (4, 7 and 8) of Au^3+^ solutions were performed to select the best concentration and pH combination for rapid GNP synthesis. The extraction of GNPs was performed by sonication of nanoparticle-loaded biomass with 7.5 mM sodium citrate solution using a Hielscher UP100H ultrasonic processor (Teltow, Germany) for 15 min at 60% amplitude, followed by centrifugation at 3000 rpm for 5 min according to the protocol of Parial et al. (2012) [5]. The extracted suspension was subsequently utilized for the characterization of GNPs.

### 2.3. Characterizations of GNPs

The maximum absorbance of synthesized GNPs was analyzed with a Hach DR6000 Benchtop UV-visible spectrophotometer (Hach, Loveland, CO, USA) in the wavelength range 300–1100 nm. A drop of GNP suspension was dried on a carbon-coated copper grid, and the size-shape analysis was carried out by a Hitachi STEM S5500 (Hitachi, Tokyo, Japan). The EDAX study was performed using the same grid and the same microscope (Hitachi STEM S5500) attached with EDAX to understand the purity of the particles.

### 2.4. Microscopic Examination of GNP Loaded N. shiloi

The appropriately dried GNP-loaded cells of *N. shiloi* were chromium coated, and the surface of the diatom cells was scanned using a Hitachi SU8020 (Hitachi, Tokyo, Japan) scanning electron microscope. The change of fluorescent property of gold-loaded cells was observed by an Axioscope A1 Zeiss fluorescence microscope (excitation: 450–490 nm and emission: 515 nm).

### 2.5. Quantification of Chlorophyll, Carotenoids and Fucoxanthin

Different biochemical parameters such as chlorophyll, total carotenoids and fucoxanthin were measured following the standard methods of Arnon (1949) [42], Carreto (1977) [43] and Wang et al. (2018) [41], respectively, in gold-treated *N. shiloi* at different time intervals (30 min, 1 h, 3 h, 12 h and 24 h). The bioactive compounds, chlorophyll and total carotenoids were extracted from 500 mg of fresh weight of *N. shiloi* using 80% pre-chilled analytical grade acetone. Fucoxanthin was extracted from the same amount of *N. shiloi* using 70% analytical grade ethanol as ethanol showed best result in fucoxanthin extraction. All the experiments were performed in triplicates. A control set was maintained for each experiment.

## 3. Results

### 3.1. Diatom Assisted Biogenesis of GNP

**Figure 1:**
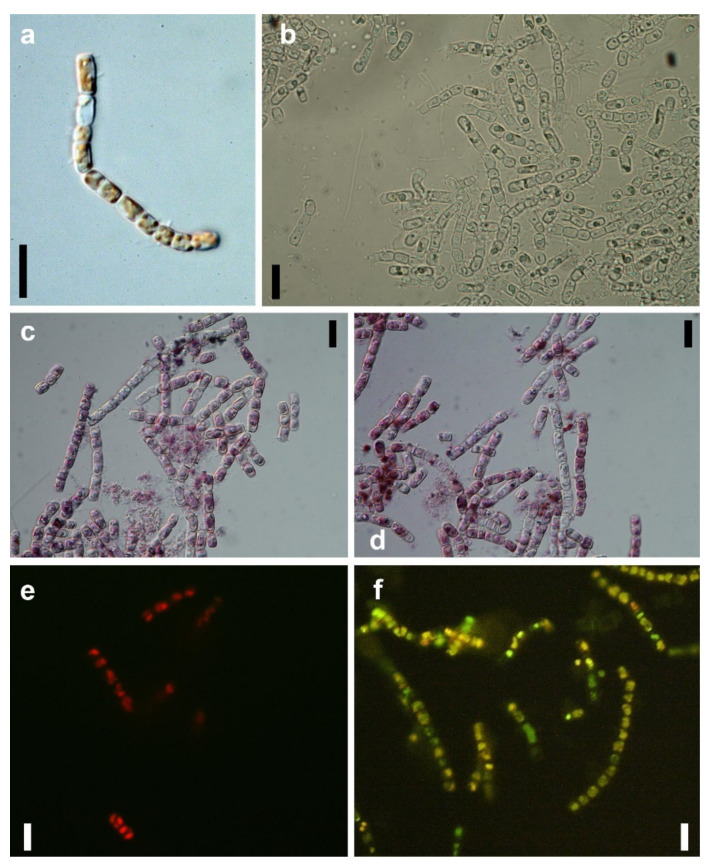
Images of a control (**a**) and gold-treated *N. shiloi* captured after 30 min (**b**), 24 h (**c**) and 72 h (**d**) of the reaction with HAuCl_4_. Fluorescent images of control (**e**) and GNP-loaded (**f**) *N. shiloi* captured in the blue light region (~450–490 nm) (Scale bars 10 µm).

The brown biomass of *N. shiloi* showed a time-dependent color change due to reaction with 25 mg L^−1^ (pH 4) Au^3+^ solution. The brown-colored cells (Figure 1a) turned green in color after 30 min of reaction due to excess production of chlorophyll (Figure 1b). The initial increase in chlorophyll production indicated the hormetic response of *N. shiloi* against gold stress. After 24 h of exposure, the appearance of pink color in diatom cells was observed (Figure 1c). The whole biomass turned pink in color after 72 h of reaction (Figure 1d). The rapid synthesis of nanoparticles was observed in reaction with 25 mg L^−1^ Au^3+^ solution at pH 4, and this condition was further used for characterization. The other concentrations (5 and 15 mg L^−1^) and higher pH (7 and 8) also showed positive responses in the synthesis of gold particles, but the reaction procedure took a much longer time (15–25 days) (Table 1). Higher concentration (35 mg L^−1^) showed lethality. Acid-washed frustules from dead cells of diatoms did not show any response in Au^3+^ reduction.

### 3.2. Morphological Changes in Gold Exposed N. shiloi

**Figure 2:**
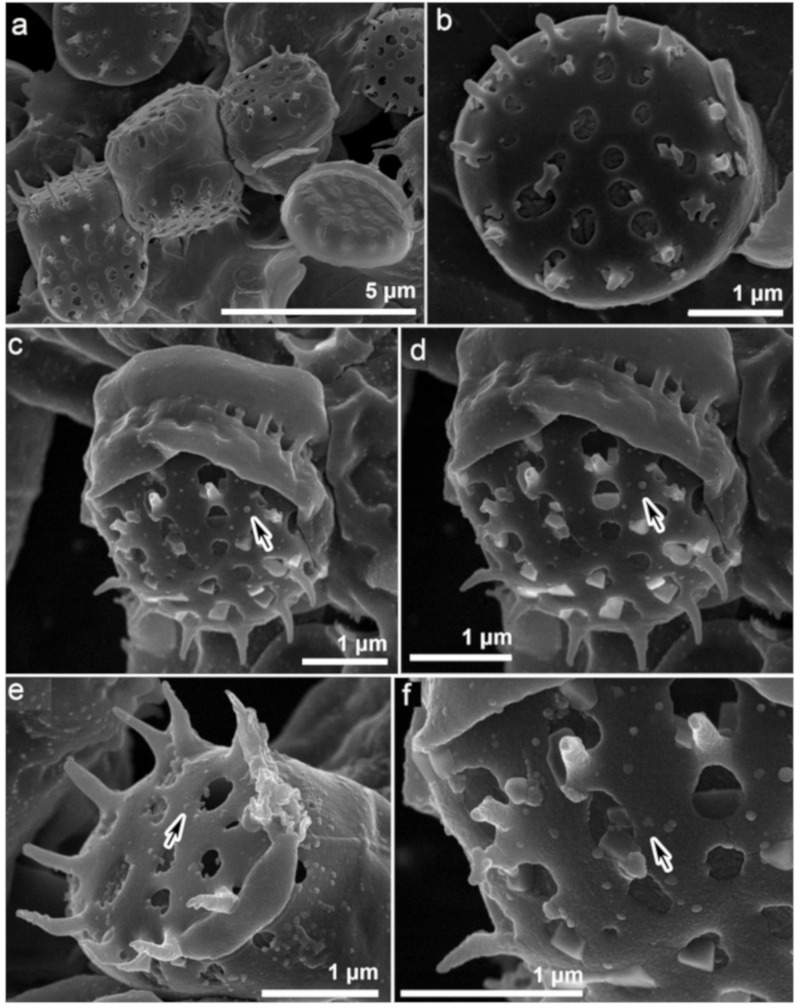
SEM images showing smooth surface topography of control cells of *N. shiloi* (**a**,**b**) and deposition of GNPs on Au^3+^-treated diatom cell surface (**c**–**f**). Arrows showing the formation of spherical (**c**,**d**) and triangular (**e**,**f**) GNPs.

The associated morphological changes in *N. shiloi* due to gold stress were documented (Figure 1a–f). During GNP production in diatom cells, the loss of chlorophyll and a high rate of cell division was observed. Under a fluorescent microscope in the blue light region (450–490 nm), gold-loaded cells of *N. shiloi* showed green fluorescence after 3 h of Au^3+^ exposure in contrast to the red fluorescent property of control cells. After 24 h, only the green fluorescence remained with greater intensity (Figure 1e,f). The SEM micrographs revealed the surface topography of control (Figure 2a,b) and gold-treated diatom frustules (Figure 2c–f). The surfaces of metal-treated frustules were fully covered with nanoparticles, which contrasts the smooth surface topography of control frustules with normal structural ornamentation. The formation of triangular and spherical-shaped GNPs was identified by SEM microphotographs (Figure 2c–f).

### 3.3. Confirmation of Au^3+^ Reduction by Isolated Fucoxanthin

**Figure 3:**
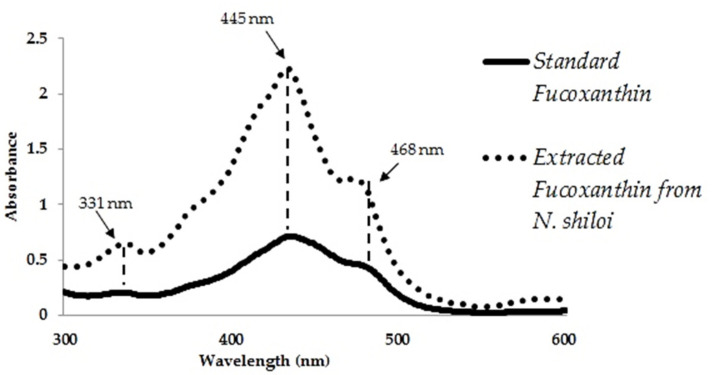
UV-vis spectroscopy of extracted fucoxanthin from *N. shiloi* and standard fucoxanthin.

The presence of fucoxanthin in ethanol extracts of *N. shiloi* was confirmed by recording the same peak positions with standard fucoxanthin in UV-vis spectroscopy (Figure 3). In UV-vis spectroscopy, the ethanol extracts of *N. shiloi* showed three distinct peaks at 331, 445 and 468 nm, respectively, as also exhibited by standard fucoxanthin. The light yellow-colored solution of HAuCl_4_ (25 mg L^−^^1^) turned pink in color within 12 h of reaction with fucoxanthin extracted from *N. shiloi* (Table 1). The appearance of pink color in HAuCl_4_ solution corroborated the ability of isolated fucoxanthin in Au^3+^ reduction and the consequent production of GNPs. The standard fucoxanthin solution also showed the same result while used as a reducing agent. Fucoxanthin showed maximum efficiency in the biofabrication of GNPs under dark conditions at pH 6 as it is sensitive to low pH and light exposure.

### 3.4. Characterization of Biogenic GNPs

**Figure 4:**
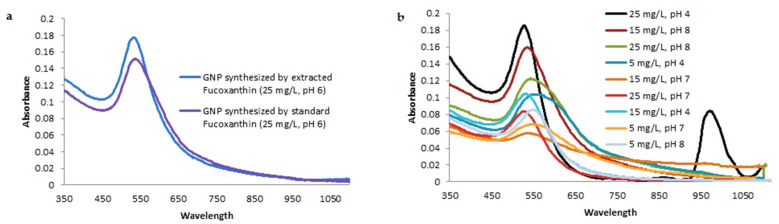
UV-vis spectroscopy of GNP synthesized by extracted fucoxanthin from *N. shiloi* and standard fucoxanthin (**a**). UV-vis spectroscopy of GNP synthesized by the whole biomass of *N. shiloi* using different concentrations and pH of HAuCl_4_ solutions (**b**).

**Figure 5:**
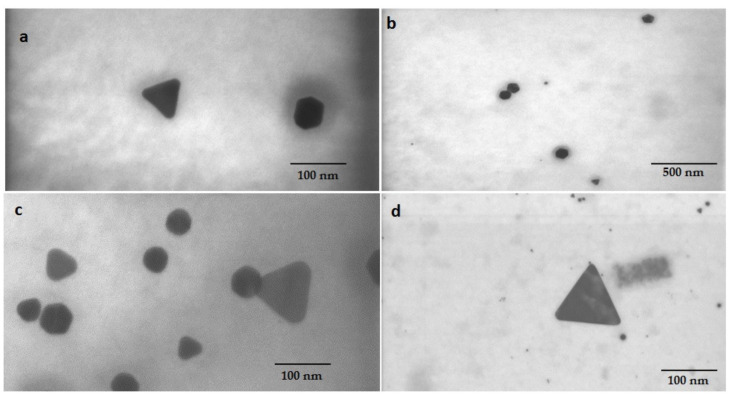
Triangular-, spherical- and hexagonal-shaped GNP synthesized by *N. shiloi*. TEM images (**a**–**d**) confirming the production of variable shaped GNPs by the whole biomass of *N. shiloi*.

**Figure 6:**
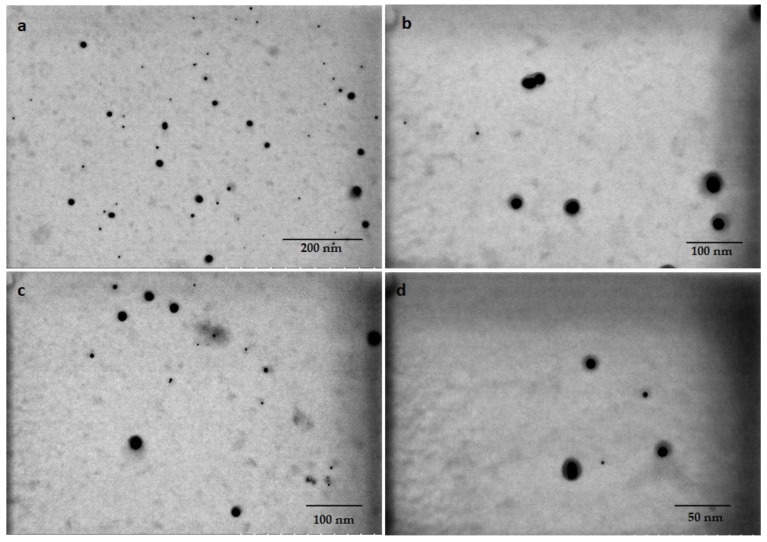
Spherical-shaped GNP synthesized by standard fucoxanthin (**a**) and extracted fucoxanthin (**b**–**d**) from *N. shiloi*.

**Figure 7:**
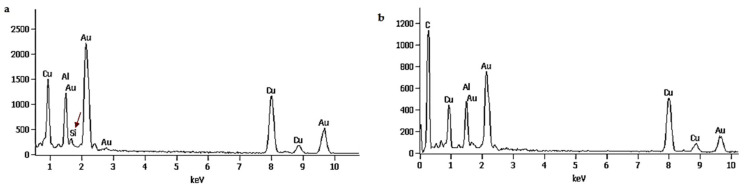
EDAX study of GNPs biofabricated by the whole biomass of *N. shiloi,* showing the presence of silica with biogenic GNPs (**a**). EDAX study of GNP produced by extracted fucoxanthin from *N. shiloi,* showing only Au signals and confirmed the absence of silica particle (**b**).

The pink suspensions synthesized by extracted and standard fucoxanthin showed distinct plasmon bands at ~525–535 and ~529–540 nm, respectively, in UV-vis spectroscopy (Figure 4a). The optical absorptions of GNPs synthesized by whole biomass of *N. shiloi* at 25 mg L^−^^1^ concentration and pH 4 of HAuCl_4_ solutions were recorded at 540 and 966 nm (Table 1, Figure 4b). Two significant bands in UV-vis spectroscopy revealed the presence of differently shaped particles. The GNPs, produced by the whole biomass of *N. shiloi* and extracted fucoxanthin did not show any measurable peak shift in UV-vis spectroscopy up to one month. The TEM studies represent a clear picture regarding the shape and size of GNPs produced by the experimental taxa. TEM micrographs of diatom cell assisted biogenic GNPs showed the synthesis of triangular-, spherical- and hexagonal-shaped particles, with triangular being the most common. The size of the particles ranged from 5 to 55 nm (Figure 5). TEM images of GNPs synthesized by extracted fucoxanthin confirmed the production of spherical-shaped particles with a 2–35 nm diameter range (Figure 6b–d). TEM micrograph revealed that GNPs synthesized by standard fucoxanthin are also spherical in nature with a 5–20 nm size range (Figure 6a). EDAX study confirmed that the GNPs synthesized by the whole biomass of *N. shiloi* are mixed with silica nanoparticles (Figure 7a), whereas the fucoxanthin-mediated biogenic particles are pure without any contamination.

### 3.5. Variation in Chlorophyll, Carotenoids and Fucoxanthin Content in GNP Loaded N. shiloi

**Figure 8:**
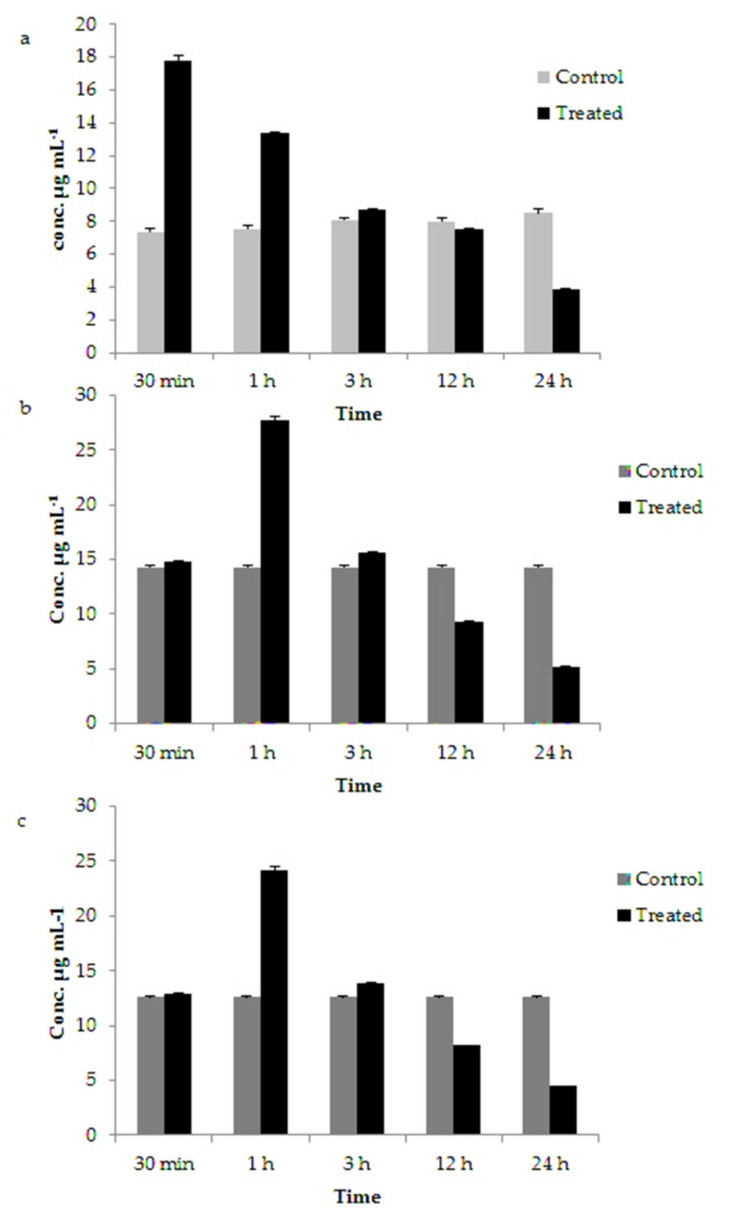
Variations in chlorophyll (**a**), carotenoids (**b**) and fucoxanthin (**c**) content in gold-treated *N. shiloi* at different time points such as 30 min, 1 h, 3 h, 12 h and 24 h.

During the synthesis of GNP, *N. shiloi* showed a significant increase in chlorophyll, carotenoid and fucoxanthin content within 1 h of reaction in comparison to control (Figure 8). The chlorophyll content in gold treated *N. shiloi* increased up to ~two times than the control after 30 min of reaction and gradually decreased thereafter (Figure 8a) (Table 2). After 72 h of reaction, there was no chlorophyll content in metal-treated *N. shiloi*. Similarly, a two-fold increase in the total carotenoids and fucoxanthin content in gold-treated *N. shiloi* were also recorded after 1 h of reaction in relation to the control sample (Figure 8b,c) (Table 2).

## 4. Discussion

It is well known that the color of the nanogold varies from light pink to purple depending upon the concentration and shape of the particles [24]. Initially, the ability of fucoxanthin in GNP production was confirmed by observing the color change of auric chloride (HAuCl_4_) solution from light yellow to pink [38]. Similarly, the pink coloration in the brown mass of *N. shiloi* was observed due to the reduction of Au^3+^ to Au^0^ and subsequent production of Au particles at the intracellular level [14]. Fucoxanthin-mediated GNP synthesis offers the benefit of producing large quantities of nanomaterials within a short time (12 h) and in a comparatively pure state. There was no contamination of silica particles, as confirmed by the EDAX study. Moreover, the extraction step can be skipped in pigment-based GNP production, whereas nanoparticles synthesized inside/surface of the cell must be isolated using additional steps such as ultrasonication [44,45]. Therefore, fucoxanthin can easily convert the downstream processing of the biofabrication of GNPs into a simple, cheap and time-saving technique.

Gold particles exhibit optical properties due to surface plasmon resonance of conduction electrons, and the color may vary from rich red to blue, depending upon the shape and size of the particles. The surface plasmon band for spherical-shaped GNPs usually has a range of 500–550 nm in aqueous solutions [46]. In our study, the red-colored GNP suspension synthesized by fucoxanthin provided a significant spectroscopic absorbance band at ~525–535 nm, indicating a nearly spherical-shaped GNP production, also confirmed by the TEM study. It was observed by Altaf and Jaganyi in 2015 [47] that triangular nanoparticles of gold exhibit two characteristic absorption bands in UV-vis spectroscopy at 540 and 966 nm attributed to plasmon resonance of the transverse and longitudinal surfaces. In the present investigation, GNP biofabricated by *Nanofrustulum shiloi* also exhibited two distinct peaks at 540 and 966 nm in UV-vis spectroscopy and indicated the presence of triangular-shaped GNP within the nanosuspension. This was also confirmed by TEM micrographs. No notable peak shift of GNPs produced by the whole biomass of *N. shiloi* and extracted fucoxanthin in UV-vis spectroscopy confirmed the stability of nanoparticles.

It is reported by many authors that carotenoids are degraded easily by environmental factors such as temperature, light radiation and oxidizing agents [48,49]. Fucoxanthin is sensitive to heat, light exposure and low pH [49]. Factors such as light promote trans-cis isomerization reactions and eventually lead to the fading color of the pigment [49]. Besides, it might also be accompanied by the formation of new compounds due to oxidation, which could give rise to the loss of color [50] and, in some cases, the formation of aroma compounds as well [48]. According to Arita et al. (2005) [50], light is a potential reason for the degradation of carotenoids during storage, as the photostability of the pigment is very much affected by its structure. Therefore, the dark conditions were maintained throughout the experiment to complete the reduction process. Fucoxanthin is less stable in acidic conditions compared to neutral and alkaline conditions [49]. Thus, pH 6 of the experimental media was maintained while using fucoxanthin as a bioreagent during GNP production. However, pH 4 gave the best result while using whole biomass as a reducing agent. Silica nanoparticles are continuously formed from monosilicic and disilicic acid within a diatom cell in silica deposition vesicles, which are acidic in nature [51]. It can be hypothesized that under acidic conditions, diatom cells produce more extracellular proteins, polysaccharides and organic acids that are able to reduce reaction time. In our experiments, a pH of 4 also showed a faster reduction of Au^3+^ and the consequent production of GNPs.

The TEM studies revealed heterogenous GNP formation by the whole biomass of *N. shiloi* due to the collective action of different reducing agents [14]. The formation of different shapes such as triangular, spherical and hexagonal are because of different reducing agents such as proteins, polysaccharides, carotenoids present in whole biomass. Production of variable shapes and sizes during biogenesis is very common as all reducing agents work together. In this study, it was observed that the production of only spherical-shaped particles is possible by using extracted fucoxanthin as a bioreagent.

The frustules of diatoms have specific sizes and shapes, as well as a unique ultrastructural pattern, by which the taxonomic enumeration of diatoms is also possible. The perforations of frustules are used for the transportation of water and minerals, by which intracellular GNPs are also transported and deposited on the surface, as observed in SEM images [19]. In gold-treated cells of *N. shiloi*, the absence of red fluorescence confirmed the non-existence of chlorophyll, whereas the presence of green fluorescence emission corroborated the green fluorescent property of nanosilica under the blue excitation at ~450–490 nm [25].

The initial increase in chlorophyll content in gold-treated *N. shiloi* was a hormetic response against gold stress, which is a common stress response in diatoms. A gradual decrease in chlorophyll content indicated no chlorophyll production. The inhibition of chlorophyll biosynthesis by metal treatment was well described by De Filippis and Pallaghy (1976) [52] and De Filippis et al. (1981) [53]. Metals interfere with the SH group of the enzymes in the chlorophyll biosynthetic pathway. Two key enzymes, d-aminolevulinic acid (ALA)-dehydratase (EC 4.2.1.24) and protochlorophyllide reductase of the chlorophyll biosynthetic pathway, were sensitive to metals. For these reasons, metal-induced inhibition of chlorophyll synthesis was reported by many authors. Gold-induced chlorophyll loss was observed in this study as well [54]. A significant increase in the total carotenoid content during metal treatment indicated active protection against Au^3+^ stress. Carotenoids serve as antioxidants against free radicals and photochemical damage [55], therefore more active against oxidative stress. It is known that there are a number of carotenoid fractions present in algal cells that are either primary or secondarily synthesized to give protection to the cells against stressed conditions [55]. Fucoxanthin—a major group of carotenoids—is responsible for energy transfer to chlorophyll a in diatoms [33]. Sliwka et al. (2007) [56] reported that in the presence of water, the hydrophilic nature of fucoxanthin enhances its electron transferability. In our study, the total experiment was performed in an aqueous-based medium that also helped fucoxanthin in transferring electrons to Au^3+^. The antioxidant- and electron-donating properties of fucoxanthin make it suitable for prompt Au^3+^ reduction and subsequent GNP production [38].

## 5. Conclusions

It can be concluded that, in this study, an eco-friendly, cost-effective, simple and rapid technique has been described to synthesize diatom-assisted GNPs. One of the smallest diatom species, *N. shiloi*, has been characterized as a potential bioreagent for the production of biocompatible, triangular GNPs within 72 h of reaction with Au^3+^ solution. The biogenic triangular particles are associated with nanosilica and can be used in cancer hyperthermia in future. The GNP decorated frustules of *N. shiloi* would be useful for label-free visualization in imaging, as nanosilica has its own fluorescent property. Brown-colored *N. shiloi* showed a hormetic response against Au^3+^ stress by producing excessive chlorophyll. It can be said that *N. shiloi* can easily interact with metals, and this strain can be used for water purification and water quality assessment. The extracted fucoxanthin from *N. shiloi* showed efficacy in synthesizing spherical shaped nanogold in isolated conditions. In this study, the accessory photosynthetic pigment, fucoxanthin, mostly available in brown algae, has been identified as a significant reducing agent in the diatom-based production of nanoparticles.

## Figures and Tables

**Table 1:** The reaction time of GNP formation in different concentrations and pH combinations of the Au^3+^ solution.

Reducing Agents	Concentration of Au^3+^ Solution (mg L^−^^1^)	pH	Reaction Time	Maximum Absorbance (nm)
Whole biomass of *N. shiloi*	5	4	20 days	~550
		7	25 days	~550
		8	22 days	~550
	15	4	7 days	~536
		7	15 days	~550
		8	10 days	~540
	25	4	3 days	~530 and ~966
		7	7 days	~535
		8	5 days	~539
Extracted fucoxanthin from *N. shiloi*	25	6	12 h	~535
Standard fucoxanthin	25	6	12 h	~537

**Table 2:** The variation in chlorophyll, carotenoids and fucoxanthin content (µg mL^−1^) in 25 mg L^−^^1^ Au^3+^ treated *N. shiloi* in different exposure times as compared to control (Maximum values (mean ± SE) are indicated as bold and italic).

Parameters	Experimental Condition	Time of Exposure				
		30 min	1 h	3 h	12 h	24 h
Chlorophyll (µg mL^−1^)	Control	7.333 ± 0.202	7.543 ± 0.223	8.066 ± 0.132	8.01 ± 0.223	8.48 ± 0.245
	25 mg L^−^^1^ Au^3+^ treated	***17.759 ± 0.329***	13.354 ± 0.057	8.682 ± 0.045	7.554 ± 0.040	3.847 ± 0.083
Carotenoids (µg mL^−1^)	Control	14.296 ± 0.169	14.396 ± 0.078	14.55 ± 0.101	14.626 ± 0.172	14.643 ± 0.189
	25 mg L^−^^1^ Au^3+^ treated	14.762 ± 0.048	***27.683 ± 0.386***	15.66 ± 0.069	9.305 ± 0.071	5.194 ± 0.042
Fucoxanthin (µg mL^−1^)	Control	12.628 ± 0.022	12.680 ± 0.038	12.748 ± 0.104	12.767 ± 0.123	12.771 ± 0.126
	25 mg L^−^^1^ Au^3+^ treated	12.94 ± 0.039	***24.099 ± 0.430***	13.878 ± 0.040	8.175 ± 0.043	4.481 ± 0.014
